# Editorial: Understanding Selfies

**DOI:** 10.3389/fpsyg.2018.00044

**Published:** 2018-02-02

**Authors:** Nicola Bruno, Katarzyna Pisanski, Agnieszka Sorokowska, Piotr Sorokowski

**Affiliations:** ^1^Unità di Neuroscienze, Dipartimento di Medicina e Chirurgia, Università di Parma, Parma, Italy; ^2^Institute of Psychology, Uniwersytet Wroclawski, Wroclawski, Poland; ^3^School of Psychology, University of Sussex, United Kingdom; ^4^Department of Psychotherapy and Psychosomatic Medicine, TU Dresden, Dresden, Germany

**Keywords:** selfies, Human Computer Interaction (HCI), internet, social media, facebook, twitter, Tinder, smartphones

With the advent of smartphones and of wide-spread Internet, every day thousands of us are taking and sharing photographic self-portraits (“selfies”) for multifarious purposes. As stated by *New York Magazine* critic Jerry Saltz (2014), selfies “have changed […] public behavior. It's become a new visual genre […] This is a very big deal for art.” In launching a research topic on selfies, we were motivated by the belief that selfies are indeed a big deal, not only for art but also for psychology. The opportunity does not come often, for a student of the human mind, to observe a brand new form of social behavior spanning issues in action, perception, cognition, personality, motivation, and social interaction. Yet, selfies have received relatively little attention by the scientific community and especially within experimental psychology. On December 1st, 2016 (before the publication of papers in this research topic), searching for the keyword “selfie” on *Google* yielded about 400 million hits, but only a meager 22 hits on the specialist database *PubMed*. We attempted to stimulate contributors by listing a series of questions that, to our minds, were worth exploring. What are the motivational, functional, and social factors driving selfie taking and posting? Are there differences between genders, age groups, ethnicities, or cultures? What biological, perceptual, cognitive, and sensorimotor factors affect selfie-taking? How might we use selfies as media tools, as sources of psychological data, or as instruments for assessing personality, stereotypes, or cultural norms? What kind of psychological object is a selfie? How does it relate to painted self-portraits, to mirror images of ourselves, and/or to our body image? Some answers to these questions are provided in this collection. The answers are partial, but interesting. It is our hope that they will stimulate further research in this area.

Theme 1. What are selfies? Do they differ from traditional photographic or painted self-portraits?

Kozinets et al. tackled these questions by focusing on the museum selfie phenomenon. Their results emphasize how the practice of selfies appears to go well beyond the mere narcissistic exhibition of the self, documenting the different modalities of communication and the different communicated contents. In a comprehensive historical review, Carbon sought to put contemporary selfies into the context of historical painted and photographic self-portraiture. He suggests that both selfies and traditional self-portraits reflect a desire to maintain and document some aspect of one's life, which, as also highlighted by Kozinets et al. may not necessarily be a narcissistic self-referential act, but rather a complex and rich means of communicating one's inner state.

Theme 2. Does the practice of selfie-taking follow specific compositional principles?

Exploiting a dataset of selfies posted on the dating application *Tinder*, Sedgewick et al. explored one aspect of a selfie's composition – whether a selfie shows the face as seen from above or below the subject. Their results reveal a systematic difference between men's and women's selfies. In selfies that did not present a neutral, frontal view (these were about 50%), men were more than twice as likely to orient the camera from below the face, whereas women tended to prefer orienting the camera from above. Manovich et al. explored another aspect of composition, namely, the choice of horizontal camera position in relation to the subject, which affects whether the selfie presents the face frontally or in three quarter pose. Exploiting a large database of selfies posted on the social network *Instagram*, from six world cities, Manovich et al. report a left cheek bias for the latter, confirming previous reports (Bruno and Bertamini, 2013; Bruno et al., 2015, 2017). In addition, they report higher expressiveness scores in selfies presenting the left cheek, in comparison to the right, for negative, but not for positive emotions. The left cheek bias in selfies was further explored by Lindell. Perusing the social network *Instagram*, Lindell identified 200 users who each posted 10 different selfies and then evaluated intra-individual consistency in selfie posing. Results indicate that selfie takers tend to adopt a preferred pose consistently, with more participants showing an overall left cheek bias in comparison to right. Finally, using computer-graphics techniques, Schneider and Carbon selected 3D face scans of 14 human models and presented them from seven camera perspectives in an online study. Although not obtained from real selfies, their results nonetheless report several potentially generalizable effects on ratings of features such as facial attractiveness, helpfulness, sympathy, dominance, distinctiveness, and intelligence.

Theme 3. Why do people take and post selfies?

Baiocco et al. tackled this question by asking participants to fill out questionnaires assessing personality structure. They report effects of sex, age, sexual orientation, and of various personality traits on selfie posting frequency. Further, analyses by Etgar and Amichai-Hamburger revealed distinct selfie-taking motivations (self-approval, belonging, and documentation) that may be differently related to personality characteristics. In combination with previous studies (e.g., Sorokowska et al., 2015; Sorokowski et al., 2015), these findings contribute to our understanding of factors motivating selfie-posting and underscore the importance of understanding selfies as a multidimensional phenomenon. Karwowski and Brzeski explored relationships between creativity and selfie posting in a large sample of *Facebook* users. Creative people were more likely to post selfies, but only if their intelligence scores were in the lowest three quartiles. Interestingly, intra-individual variation in selfie-posting increased when participants engaged in creative activities, such as painting or blogging (but not science-related activities), such that the variability in selfie-posting within-individuals more than doubled the differences between them. This work suggests that transient, situational factors may be more important than stable personality traits for understanding selfies. Finally, Dhir et al. addressed a lesser studied deterrent to selfie posting—concern over personal privacy. Privacy concerns correlated with a reduction in selfie behaviors among adult males and females of all ages, but not male adolescents and male young adults. These findings have implications for theories of selfie-related behaviors, but also for policy makers.

Theme 4. Does the format or content of selfies provide cues to personality or otherwise provide information about the taker?

Musil et al. explored how visual cues in selfies may reveal personality characteristics of the selfie-takers. Despite obtaining complex results, these authors failed to identify systematic visual cues to personality characteristics of selfie takers. If their results will be confirmed in later contributions, this will lead to the conclusion that the use of selfies in personality assessment is limited. Krämer et al. examined how viewers evaluate selfies in comparison to regular photographic portraits. Using *Facebook* profile mockups that were either selfies or regular photographs, of female or male subjects, and that included either one individual or groups, they found that selfie takers were rated as less trustworthy, less socially attractive, less open to new experiences, more narcissistic, and more extroverted in comparison to the same individuals when featured in regular photographs. This suggests that selfies may evoke negative assessments more often than selfie takers presuppose. Diefenbach and Christoforakos conducted an online survey assessing selfie-related behaviors. Their results reveal a systematic discrepancy between attitudes to one's own selfies. While own selfies were rated more positively than other's selfies, participants reported more negative consequences of selfies than positive, and had a strong preference for viewing more usual pictures in comparison to selfies on social media. This is an intriguing paradox: respondents claim they dislike selfies, and yet provide justifications for their own selfie-taking. These findings call for more research on social attitudes toward selfies and related mechanisms.

## Author contributions

Conceived and designed the Research Topic: PS, AS, KP, and NB; Wrote the Editorial: NB, KP, PS, and AS.

### Conflict of interest statement

The authors declare that the research was conducted in the absence of any commercial or financial relationships that could be construed as a potential conflict of interest.

## References

[B1] Bruno N. Bertamini M. (2013). Self-portraits: smartphones reveal a side bias in non-artists. PLoS ONE 8:e55141. 10.1371/journal.pone.0055141 23405117 PMC3566189

[B2] Bruno N. Bertamini M. Protti F. (2015). Selfie and the City: a World-Wide, Large, and ecologically valid database reveals a two-proged side bias in naive self-portraits. PLoS ONE 10:e0124999. 10.1371/journal.pone.0124999 25915767 PMC4411109

[B3] Bruno N. Bode C. Bertamini M. (2017). Composition in portraits: selfies and wefies reveal similar biases in untrained modern youths and ancient masters. Laterality 22, 279–293. 10.1080/1357650X.2016.1185108 27229630

[B4] Saltz J. (2014). Art at Arms' Length: A History of the Selfie. New York Magazine, 47, 71–75.

[B5] Sorokowska A. Oleszkiewicz A. Frackowiak T. Pisanski K. Chmiel A. Sorokowski P. (2015). Selfie and personality: who posts self-portrait photographs. Pers. Individ. Dif. 90, 119–123. 10.1016/j.paid.2015.10.037

[B6] Sorokowski P. Sorokowska A. Oleszkiewicz A. Frackowiak T. Huk A. Pisanski K. (2015). Selfie posting behaviors are associated with narcissism among men. Pers. Individ. Dif. 85, 123–127. 10.1016/j.paid.2015.05.004

